# Recombination of Human Coxsackievirus B5 in Hand, Foot, and Mouth Disease Patients, China

**DOI:** 10.3201/eid1802.111524

**Published:** 2012-02

**Authors:** Jian-Feng Han, Tao Jiang, Xing-Liang Fan, Li-Ming Yang, Man Yu, Rui-Yuan Cao, Jun-Zhi Wang, E-De Qin, Cheng-Feng Qin

**Affiliations:** State Key Laboratory of Pathogen and Biosecurity, Beijing, People’s Republic of China (J.-F. Han, T. Jiang, L.-M. Yang, M. Yu, R.-Y. Cao, E.-D. Qin, C.-F. Qin);; Beijing Institute of Microbiology and Epidemiology, Beijing, People’s Republic of China (J.-F. Han, T. Jiang, L.-M. Yang, M. Yu, R.-Y. Cao, E.-D. Qin, C-.F. Qin);; National Institutes for Food and Drug Control, Beijing (X.-L. Fan, J.-Z. Wang)

**Keywords:** human enterovirus B, genetic recombination, phylogeography, coxsackievirus, viruses, HFMD, China, hand, foot, and mouth disease

**To the Editor:** Hand, foot, and mouth disease (HFMD) is an acute viral infectious disease in infants and young children. However, since 2008, HFMD has emerged as a major public health problem in the People’s Republic of China, resulting in millions of infections with hundreds of deaths (*1*). Human enteroviruses (HEVs), including HEV71, echoviruses, and coxsackie viruses A and B (CAV and CBV), are the major pathogens of HFMD (*2*). In mainland China, HEV71 and CAV16 have been recognized as the dominant causative agents for HFMD.

During a recent HFMD outbreak in Changchun during 2010, three of 16 throat swab samples tested positive for HEV but negative for HEV71 and CAV16 by reverse transcription PCR. All 3 samples were then placed into human rhabdomyosarcoma cells, and typical cytopathic effects were observed 3–4 days later. All the isolates were finally characterized as CBV5 by using serologic and molecular technology and designated as CBV5/CC10/10, CBV5/CC10/16, and CBV5/CC10/17, respectively. The complete genome of these Changchun isolates was determined as described (*3*) and submitted to GenBank (accession nos. JN580070, JN695050, and JN695051, respectively. The genome RNA of CBV5/CC10/10 is 7,402 bp long, and the 5′ and 3′ untranslated regions are 743 and 101 bp, respectively. The coding regions of these Changchun isolates are highly homologous, with amino acid identity of 100% and only a 3-nt difference exists among them. The complete genome of 4 CBV5 strains were indexed previously in GenBank, and the nucleotide sequence identities of the newly isolated CBV5/CC10/10 with the other 4 CBV5 strains were among 80.6%–88.1%. Results of homology and phylogenetic analyses based on the complete viral protein 1 sequence (849 bp) showed that the nucleotide identity of viral protein 1 among 17 different CBV5 strains was 78.9%–95.6% and the amino acid identity was 92.9%–98.9%. The neighbor-joining tree indicated that the new isolated CBV5 Changchun strains were most closely related to the strains isolated from mainland China and that they divided into a distinct lineage from other CBV5 strains outside China (Figure). CBV5 infections were reported in mainland China during 2002–2010 in Zhejiang, Shandong, and Henan Provinces (*4*). These Changchun isolates were highly homologous with the recent Henan isolate, COXB5/Henan/2010, with a nucleotide identity of 88.1%. These results indicated that CBV5 might have been circulating in China for nany years and represented an independent evolution tendency.

**Figure:**
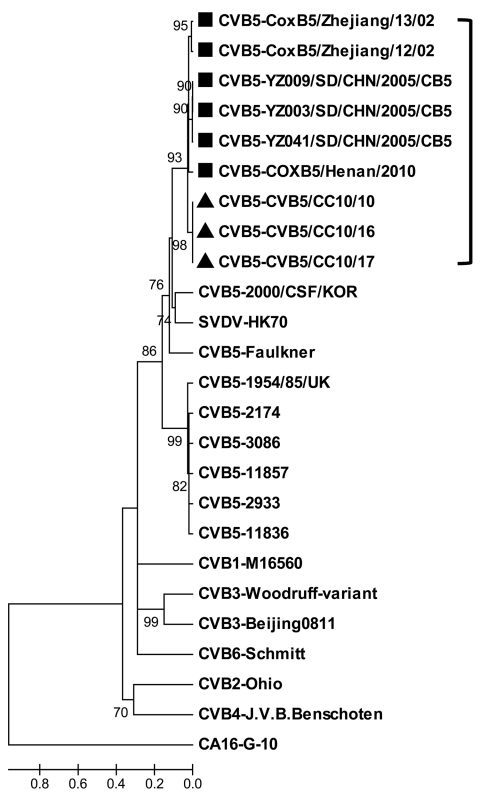
Phylogenetic analysis of selected human coxsackievirus B (CBV) strains from different origins based on the viral protein 1 gene sequences. The neighbor-joining tree was generated by using MEGA4 software (www.megasoftware.net), and the prototype strain of coxsackievirus A (CAV) 16 was used as outgroup. The Changchun strains isolated in this study are indicated by triangles and other Chinese CBV5 strains are indicated by squares. Scale bar indicates nucleotide substitutions per site.

Homology and BLAST analysis (http://blast.ncbi.nlm.nih.gov/Blast.cgi) based on the complete genome sequence showed that these newly CBV5 isolates have 85% identity with some human CBV3 strains. Because RNA recombination is a well-known phenomenon for HEVs during viral evolution and reemergence (*5**–**8*), recombination analysis between newly isolated CBV5 and other HEVs was performed by using SimPlot software. Similarity scanning analysis (Technical Appendix Figure 1) by using CBV5/CC10/10 as query sequence showed that the 5′ half (nt 1–4481) of the genome had high similarity (>93%) to CBV5 strain COXB5/Henan/2010, and the 3′ half (nt 4661–7402) showed high similarity (>97%) to CBV3 strain Beijing0811. Then, bootscanning analysis (Technical Appendix Figure 2) showed that CBV5/CC10/10 was most closely related to COXB5/Henan/2010 in the 5′ half of the genome, and to CBV3 Beijing0811 in the 3′ half of the genome. Both analyses showed that the small fragment within 2C domains are highly similar (>84%) to a CBV5 strain from South Korea, CBV5/2000/CSF/KOR. Genetic algorithms for recombination detection analysis also indicated that the putative breakpoints were located within the 2C domain. We found no recombinant evidence between CBV5 and other HEVs during the analysis. Together, these accordant results demonstrated that recombination has possibly occurred within the 2C domain, and these Changchun isolates are possibly progeny of intertypic and intratypic recombination of CBV strains circulating in China (COXB5/Henan/2010 and Beijing0811) and South Korea (2000/CSF/KOR).

HEVs can be divided into ≈100 serotypes, and intertypic or intratypic recombination among different viruses occurs frequently, which is information necessary for disease control and surveillance. In China, dozens of HEVs, including HEV71, CAV, CBV, and echoviruses, have been isolated from HFMD patients and identified as the pathogens causing HFMD (*1**,**2**,**9**,**10*). Recombination events among HEVs have been shown to play roles in HFMD outbreaks. A recombinant HEV71 with CAV16 was suggested to be responsible for the HFMD outbreak in Fuyang, China, in 2008 (*9*). Recombination also occurred among the CAV (types 2, 4, 5, and 10) isolates during a HFMD outbreak in China during 2009 (*10*). However, most recombinant studies have focused on CAV and poliovirus. Recently, Oberste et al. (*6*) proposed the possible role of recombination within CBV strains based on phylogeneic analysis of individual genes. Our current study identified and characterized the newly CBV5 isolates from HFMD patient as intratypic and intertypic recombinants, and further surveillance is warranted that focuses on the emerging recombinant viruses among HFMD causative agents and investigations on the pathogenic role and disease associations of the recombinant CBVs.

## Supplementary Material

Technical Appendix Similarity scanning and full-length bootscanning analysis of coxsackievirus B (CBV) 5/CC10/10 with other CBV strains and representative enterovirus strains.
