# Supplementary material for: Recombination of Human Coxsackievirus B5 in Hand, Foot, and Mouth Disease Patients, China
Source: Emerg Infect Dis. 2012 Feb;18(2):351–3. doi: 10.3201/eid1802.111524 (PMC3310474; doi:10.3201/eid1802.111524)
Supplement: Technical Appendix — Similarity scanning and full-length bootscanning analysis of coxsackievirus B (CBV) 5/CC10/10 with other CBV strains and representative enterovirus strains. [file 11-1524-Techapp_2p.pdf]

Article DOI: http://dx.doi.org/10.3201/eid1802.111524

Recombination of Human Coxsackievirus B5 in Hand, Foot, and Mouth Disease Patients, China

Technical Appendix

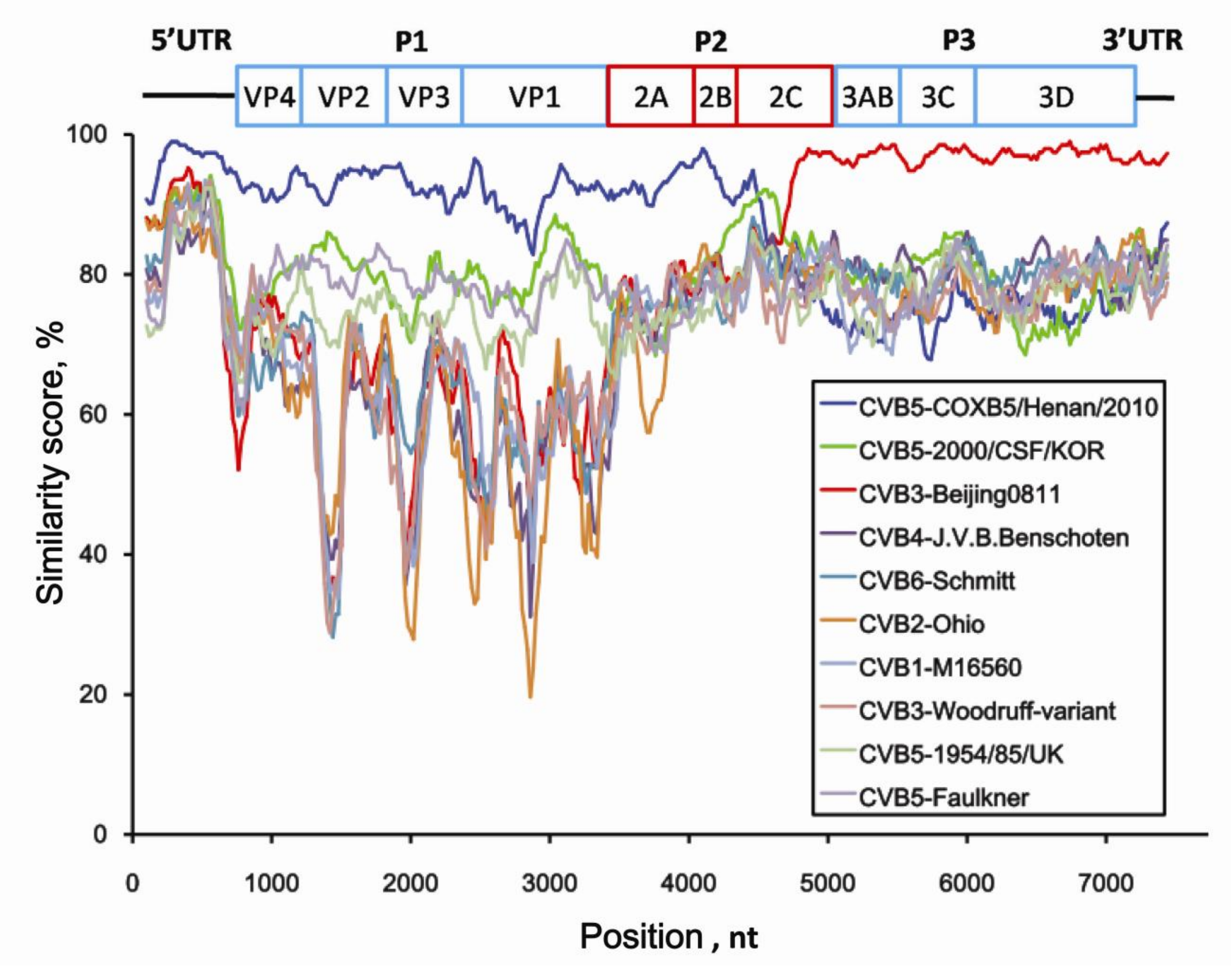

Technical Appendix Figure 1. Similarity scanning analysis of coxsackievirus B (CBV) strain CBV5/CC10/10 with other CBV strains and representative enterovirus strains on the basis of full-length genome sequences.

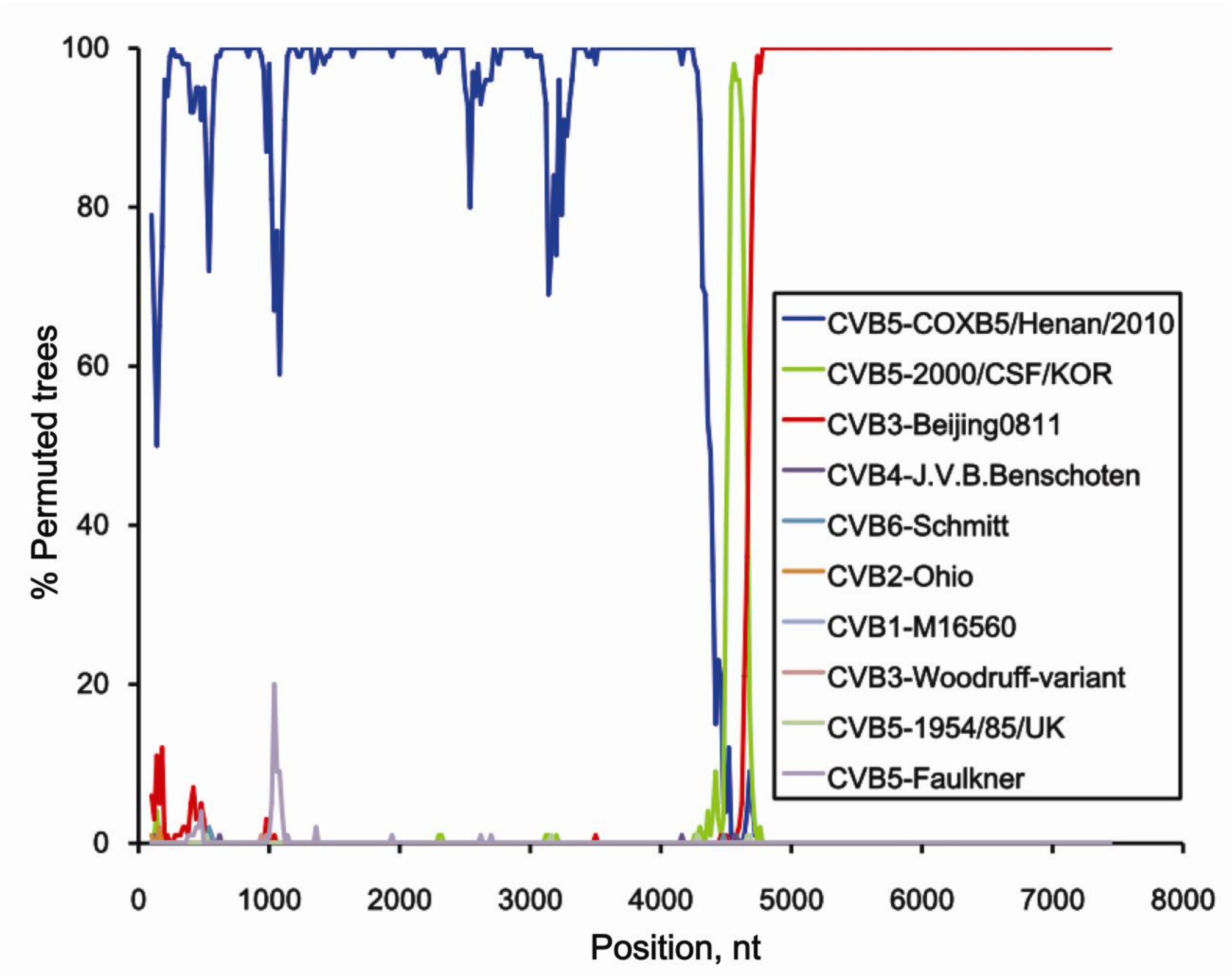

Technical Appendix Figure 2. Full-length bootscanning analysis of coxsackievirus B (CBV) strain CBV5/CC10/10 with CBV3-Beijing0811, CBV5–2000/CSF/KOR, and CBV5-COXB5/Henan/2010. Bootscanning was performed with a window size of 200 nt and step of 20 nt.
